# Evidence of a Causal Relationship Between Smoking Tobacco and Schizophrenia Spectrum Disorders

**DOI:** 10.3389/fpsyt.2018.00607

**Published:** 2018-11-20

**Authors:** James G. Scott, Lori Matuschka, Solja Niemelä, Jouko Miettunen, Brett Emmerson, Antti Mustonen

**Affiliations:** ^1^Faculty of Medicine, The University of Queensland, Herston, QLD, Australia; ^2^Queensland Centre for Mental Health Research, Wacol, QLD, Australia; ^3^Metro North Mental Health, Royal Brisbane and Women's Hospital, Herston, QLD, Australia; ^4^Faculty of Medicine, University of Turku, Turku, Finland; ^5^Department of Psychiatry, South-Western Hospital District, Turku, Finland; ^6^Center for Life Course Health Research, University of Oulu, Oulu, Finland; ^7^Medical Research Center Oulu, Oulu University Hospital and University of Oulu, Oulu, Finland

**Keywords:** schizophrenia, psychosis, nicotine, smoking, causal, association, e-cigarette

## Abstract

There has been emerging evidence of an association between tobacco smoking and schizophrenia spectrum disorders (SSD). Two meta-analyses have reported that people who smoke tobacco have an ~2-fold increased risk of incident schizophrenia or psychosis, even after adjusting for confounding factors. This study aimed to critically appraise the research which has examined the association between tobacco smoking and SSD against the Bradford Hill criteria for causality, to determine the strength of the evidence for a causal relationship. Eight longitudinal studies (seven cohort studies and one case control study) were identified which examined tobacco smoking as an exposure and psychosis as an outcome. All seven cohort studies were assessed as being of high quality using the Newcastle-Ottawa Scale. Six of the eight studies found a statistically significant positive association between tobacco smoking and onset of SSD. These studies reported a consistent association with a moderate to large effect size and a dose response relationship. The studies adjusted for multiple potential confounders including age, sex, socioeconomic status, shared genetic risk, prodromal symptoms, and comorbid cannabis and other substance use. The studies did not adjust for exposure to childhood trauma or prenatal tobacco. There was substantial though inconclusive evidence supporting a causal relationship between tobacco smoking and increased risk of SSD. If a causal relationship does exist, nicotine is most likely responsible for this association. This raises serious public health concerns about the increasing use of e-cigarettes and other products, particularly by adolescents whose nicotine use may increase their risk of SSD. Research is urgently needed to examine the association between e-cigarette use and incident psychosis, particularly in adolescents and young adults.

## Introduction

Schizophrenia spectrum disorders (SSD) are heterogeneous syndromes with well-established risk factors including exposure to childhood adversity, cannabis use during adolescence, a history of obstetric complications, stressful events during adulthood, and low maternal serum folate level (1). In recent years, there has been a growing interest in tobacco smoking as a risk factor for SSD (2, 3).

Tobacco smoking is known to cause a wide range of physical health problems. It is the leading cause of preventable death, through increasing the risk of lung and other malignancies, chronic obstructive pulmonary disease (COPD), coronary heart disease, cerebrovascular disease, asthma and diabetes (4). Two systematic reviews and meta-analyses have examined the association between tobacco smoking and psychotic disorders (2, 3). In pooling longitudinal studies (*n* = 5), Gurillo and colleagues reported a 2-fold increase in the risk of incident psychotic disorders in people who were daily tobacco smokers compared to those who were not (RR = 2.18; 95% CI 1.23–3.85). Similarly, Hunter et al. (3) who pooled data from studies identified using inclusion criteria with the outcome restricted to schizophrenia (*N* = 5) also reported smoking tobacco was associated with a 2-fold risk of schizophrenia (RR = 1.99; 95% CI 1.10–3.61). Both studies concluded that further research was needed to examine the potential causal role of tobacco smoking in the onset of SSD.

The association between tobacco smoking and SSD is of growing significance. There is evidence that nicotine alters signaling in the dopaminergic, cholinergic, and glutamatergic neurotransmitter systems, particularly in adolescence (5). Whilst the smoking of tobacco by young people has declined in many high income countries, there has been an increase in exposure to nicotine by this demographic through the availability of e-cigarettes (6). It is therefore important to critically examine the evidence for a causal relationship between tobacco smoking and SSD.

In this review we aimed to evaluate the relationship between tobacco smoking and SSD which we defined as any non-affective psychotic disorder against causal criteria based on the Bradford Hill Framework (7, 8). The Bradford Hill Framework provides nine criteria for establishing a causal relationship between an exposure and outcome. This review examined longitudinal studies identified from the two recent systematic reviews of tobacco smoking and incident SSD and other identified studies. The evidence for a causal relationship between tobacco smoking and SSD, alternative explanations for the association and the health implications are discussed.

## Methods

### Literature search

We used the results of the two recently conducted systematic reviews (2, 3) to identify studies which examined tobacco smoking as an exposure and SSD as an outcome. As the review by Hunter et al. (3) restricted the outcome to a diagnosis of schizophrenia, we used the broader search strategy of Gurillo et al. (2) to identify studies from January 2014 to May 2018 that included the broader outcome of psychosis. These psychosis outcomes included schizophrenia, schizophreniform disorder, schizoaffective disorder, delusional disorder, non-affective psychotic disorder, atypical psychosis, psychotic depression, and bipolar mania with psychotic features.

The inclusion criteria of the current review were: (a) longitudinal case control or cohort studies; (b) study populations of participants with psychosis or schizophrenia as the outcome (defined as those who meet the diagnostic criteria by structured interview or diagnosed by treating clinician); (c) presence of tobacco smoking prior to psychosis or schizophrenia diagnosis. Studies which were cross sectional in design or only provided sub-diagnostic outcomes of psychosis (e.g., psychotic symptoms, hallucinations, delusions) were excluded.

### Data extraction

Titles and abstracts of the articles were reviewed to identify studies that met the eligibility criteria. The following characteristics were extracted from each study when available: (a) study methodology (including author, publication year, location, study design, follow-up period, sample numbers, loss to follow-up, age at baseline, tobacco smoking measures, and assessment of psychosis or schizophrenia), and (b) study findings (effect size metrics, 95% CI, and confounders adjusted for).

The quality or the studies assessing for risk of bias was evaluated using Newcastle–Ottawa Scale (NOS) (9) as shown in Supplementary Table 1. The NOS is a method recommended by the Cochrane Non-randomized Studies Methods Working Group to evaluate the quality of the study. Points are assigned based on the selection process of cohorts (0–4 points), the comparability of the cohorts (0–2 points) and the identification of the exposures and the outcomes of research participants (0–3 points). A score of 7 or greater out of 9 was defined as high quality. Studies were assessed independently by two reviewers (LM and JS).

### Assessment of causality

Studies that met inclusion and exclusion criteria were assessed using causal criteria based on the Bradford Hill Framework shown in Supplementary Table 2. Of the nine criteria, five were chosen as most relevant for the purposes of this study (strength of association, consistency, temporality, dose-response, and biological plausibility). Given that smoking is known to cause a wide range of health problems, the criteria of specificity was not applicable. No studies have performed experimental manipulation exposing adolescents to tobacco because of the known harmful effects therefore this criteria was not included. Coherence was not included because of the lack of homogenous pathology evident in psychosis. In relation to analogy, the association between cannabis use and psychosis, reported to be causal (1) has some analogy to that of tobacco and psychosis. However, it is widely recognized that adolescents who smoke tobacco are more likely to smoke cannabis (10–12). Thus, cannabis rather than being analogous to tobacco in its relationship with psychosis may in fact be an important confounder. Similarly there are other important environmental factors which might confound the relationship between tobacco smoking and incident psychotic disorder. To address this concern, for the purpose of assessing evidence of causality, we included an extra criteria “accounted for confounding.” These six criteria were deemed appropriate by the research team in order to grade the associations reported between adolescent tobacco smoking and future risk of SSD as a basis for causality discussion (7).

## Results

Gurillo and colleagues (2) identified four studies which met the specified inclusion criteria (13, 14, 15, 16). One of the longitudinal studies (17) which they included in their pooled analysis did not determine the presence of tobacco smoking before the schizophrenia diagnosis and was therefore excluded. Hunter et al. (3) included another study (18) and the updated search identified a further three studies which met inclusion criteria (13, 19, 20, 21). In total, eight studies (seven cohort and one case-control studies) were included for assessment of a causal relationship between tobacco smoking in adolescence and incident SSD. Using the NOS, all seven cohort studies scored 7/9 or greater demonstrating they were of high quality (Table 1).

**Table 1:** Assessment of study quality using the Newcastle Ottowa Scale^*^.

**Criteria**	**Kendler et al. (13)**	**Mustonen et al. (21)**	**McGrath et al. (19)**	**Sørensen et al. (15)**	**Weiser et al. (14)**	**Wium-Andersen et al. (18)**	**Zammit et al. (16)**
Representativeness of exposed cohort	+	+	+	+	+	+	+
Selection of non-exposed cohort	+	+	+	+	+	+	+
Ascertainment of exposure	+	–	–	+	–	–	–
Demonstration that outcome of interest was not present at start of study	+	+	+	+	+	+	+
Comparability of cohorts on basis of design and analysis	++	++	++	+	+	+	++
Ascertainment of outcome	+	+	+	+	+	+	+
Follow-up adequate for outcome to occur	+	+	+	+	+	+	+
Adequacy of follow-up of cohorts	–	–	–	–	+	+	–
Total Score	8/9	7/9	7/9	7/9	7/9	7/9	7/9

* *A score of 7/9 or greater represents a high-quality study*.

### Study characteristics

Table 2 summarizes the study characteristics. They utilized birth cohort studies of offspring (19, 21) or mothers (15), cohorts of young male conscripts from defense forces (14, 16), two cohorts combined, the first consisting of mothers recruited from a birth cohort, the second were male conscripts Kendler et al. (13) and two general population cohorts to assess cardiovascular risk factors (18). The longitudinal case control study was of participants at clinical high risk of psychosis (20). All studies were from high income countries. The follow-up period of all cohort studies was adequate to ascertain incident cases of SSD, ranging from a minimum of 4 years (14) to a maximum of 48 years (15). Two of the studies were genetically informed with one examining psychosis risk in family members discordant for smoking (13), the other examining schizophrenia in people with different alleles of the rs1051730 genotype in the nicotinic acetylcholine receptor gene stratified by smoking status (18).

**Table 2:** Longitudinal studies examining the association between tobacco smoking and later schizophrenia and related disorders.

**Reference (Country and Methodology)**	**Sample size (psychosis prevalence)**	**Diagnoses and how they were ascertained**	**Definition of smoking (prevalence of smoking)**	**Length of follow-up**	**Results (95% CI)**	**Covariates**	**Interpretation**
Buchy et al. (20) (USA; Prospective Case-Control study)	362 Clinical High Risk participants (90 transitioned to psychosis over 2 years)	Any psychotic disorder or a rating of ≥6 on any positive symptom of Scale of Prodromal Symptoms	At least occasionally	2 years	Baseline tobacco use (frequency or severity) was not different between those participants who later transitioned to psychosis (*n* = 90) from those without transition (*n =* 272). Cannabis and alcohol use were not associated with psychosis transition.	Demographic variables, cannabis use, alcohol use, cocaine, opiates, other substances	Tobacco, alcohol and cannabis use (frequency or severity) were not associated with increased risk of transition to psychosis. The prevalence of tobacco and cannabis dependence were very low in this cohort with likely inadequate power to examine these longitudinal associations.
Kendler et al. (13) (Two Swedish Cohorts using birth and conscript registries)	1,413,849 women and 233,879 men	Schizophrenia and non-affective psychosis (ICD)	At least 1 cigarette/day (NA)	Females 18.5 years, males 7.9 years	Increased risk of subsequent schizophrenia in females who were light smokers (1–9 cigarettes/ day: HR = 2.21; 95% CI 1.90–2.56) and for heavy smokers (≥10 cigarettes/day) (HR = 3.45; 95% CI 2.95–4.03). Increased risk of subsequent schizophrenia for male light smokers (HR = 2.15; 95% CI 1.25–3.44) and for heavy smokers: HR = 3.80; 95% CI 1.19–6.60). Increased risk persisted after adjusting for covariates and was present in monozygotic twins who were discordant for smoking status.	Neighborhood and parental socioeconomic status, prior drug abuse, psychosis prodrome, family-level and community-level socioeconomic status and genetic liability to psychosis	Cigarette smoking increased the risk of schizophrenia in a dose response fashion. The association cannot be attributed to incident smoking in the prodromal phase. Accounting for genetic disposition using monozygotic twins discordant for smoking resulted in an attenuation of the association however it was still significant suggesting the relationship between smoking cigarettes and future schizophrenia risk is only partially explained by shared risk genes.
McGrath et al. (19) (Australia: Mater University of Queensland Birth Cohort (MUSP)	2,441 of whom 65 (2.6%) received a diagnosis of psychosis	Non-affective psychotic disorder based on the Composite International Diagnostic Interview	Age at first tobacco use self-reported at 21 years. Participants grouped into age of tobacco use ≤ 15 years (24.1%), 16–21 years (25.8%) and no use.	6 years	Early onset tobacco use was associated with non-affective psychosis after adjusting for age and sex (OR 3.1; 95% CI 1.8-5.6). After excluding those with a history of cannabis use, the association attenuated (OR 1.9; 95% CI 0.09–4.3).	Age, sex and cannabis use	Early onset tobacco use was associated with later psychosis. The loss of significance of this relationship after excluding those with a history of cannabis use may be attributed to a loss of power as the direction of the relationship (positive association) remained.
Mustonen et al. (21) (Northern Finland Birth Cohort; 1986)	6,081 of whom 110 (1.8%) developed psychosis	Any psychotic disorder (ICD) based on clinical diagnoses from hospital summaries, primary health care and specialists	At least 1 cigarette/day (12.3%)	14 years	Smoking 1–9 cigarettes/ day was not associated with psychosis. Smoking ≥ 10 cigarettes/day was associated with increased risk of psychosis (Unadjusted HR = 3.15, 95% CI 1.94–5.13; Fully adjusted HR = 2.00, 95% CI 1.13–3.54). A dose–response was reported with a positive trend test (fully adjusted OR = 1.05; 95% CI: 1.01–1.08).	Prodromal symptoms, Cannabis use, alcohol use, other substance use, parental substance abuse, parental psychosis	Smoking cigarettes was associated with an increased risk of psychosis after adjusting for a wide range of covariates. There was a dose response relationship between smoking cigarettes and future risk of psychosis.
Sørensen et al. (15) (Denmark; Mothers from the Copenhagen Perinatal Cohort)	7926 of whom 309 (3.9%) developed schizophrenia spectrum disorder	Diagnosis of Schizophrenia spectrum disorder (schizoaffective disorder, schizophrenia, schizophrenia-like psychosis) in the Danish National Registry	At least 1 cigarette/day (52.1%)	46–48 years	There was an association between smoking and increased risk of subsequent schizophrenia spectrum disorder: (OR and 95% CI 1.42; 1.12–1.80). There was a linear effect of smoking (1.18; 1.07–1.30).	Age, social status, psychopharmacological treatment at baseline	Cigarette smoking in women attending antenatal care increased the risk of subsequent schizophrenia spectrum disorder in a dose response fashion.
Weiser et al. (14) (Young Male Cohort (16–17 years) from the Israel Defence Force)	14,248 of whom 44 (0.3%) were hospitalized for schizophrenia	Hospitalized with an ICD 10 diagnosis of Schizophrenia	At least 1 cigarette/day (28.4%)	4-16 years (mean = 10.2 years, SD = 3.6)	Those who smoked at least one cigarette/ day were at increased risk of schizophrenia (adjusted relative risk = 1.94, 95% CI 1.05–3.58). There was a significant linear association between number of cigarettes smoked and risk of schizophrenia where smoking increased risk of subsequent schizophrenia.	Non-psychotic psychiatric disorder, below-normal social or intellectual functioning in adolescence and socioeconomic status	Cigarette smoking in young adult males increased the risk of schizophrenia. A dose response relationship was reported.
Wium-Andersen et al. (18) (Two General Danish Population Cohorts: Copenhagen General Population Study and Copenhagen City Heart Study	63,296 (0.1% hospitalized for schizophrenia and 6% had purchased antipsychotic medication)	ICD diagnoses of Schizophrenia and purchasing of antipsychotic medication obtained from the national Danish Patient Registry.	Ever smoked (63%), Cigarettes/ day and pack-years calculated from a self-report questionnaire.	3–21 years	Compared with never-smokers, participants smoking >20 cigarettes/day had an increased risk of schizophrenia (adjusted OR and 95% CI 6.18; 2.77–13.8).	Alcohol use, weekly physical activity, level of education after lower secondary school, basic vocational training, level of income, civil status, plasma levels of C-Reactive Protein and comorbid physical illness	Smoking tobacco was associated with higher risk of schizophrenia and antipsychotic medication use. The rs1051730 genotype in the nicotinic acetylcholine receptor gene was associated with psychosis outcomes in the ever smokers but not the never smokers suggesting a causal relationship between cigarettes and psychosis outcomes.
Zammit et al. (16) (Cohort of males conscripted into the Swedish army between 1969 and 1970)	50,053 of whom 363 (0.7%) were diagnosed with schizophrenia	ICD Diagnoses of schizophrenia and other psychoses extracted from the Swedish National Register of Inpatient Care	At least 1 cigarette/day (59%)	27 years	There was no association between any daily smoking and subsequent schizophrenia (adjusted Hazard Ratio (aHR) 0.8, 95% CI 0.7–1.1). Those who smoked ≥ 20 cigarettes/ day were less likely to develop schizophrenia (aHR 0.5; 95% CI 0.3–0.9) and there was a significant linear trend where smoking decreased the risk of subsequent schizophrenia (aHR 0.8; 95% CI 0.7–0.9).	Cannabis and drug use, poor social integration, disturbed behavior, IQ, place of upbringing, family economy, and family psychiatric history	After adjusting for potential confounders, there was a decrease in the risk of schizophrenia in people who smoked cigarettes. There was a dose response reduction with those who smoked the most cigarettes having the lowest risk of schizophrenia.

### Assessment of studies against bradford hill criteria

Using causal criteria, based on the Bradford Hill Framework Hill (8), of the eight studies examined, six reported a positive association between tobacco smoking and risk of schizophrenia spectrum disorder. The strength of the associations were robust ranging from an almost 50% increased risk (15) to a 6-fold increased risk of schizophrenia in heavy smokers (18). In these six studies, all reported a temporal association with appropriate adjustment for confounding variables, particularly comorbid substance use. All but one (19) demonstrated a dose response relationship between tobacco use and SSD. By contrast, one study (16) reported that smoking tobacco reduced the risk of schizophrenia and the case control study (20) found no association.

## Discussion

Two meta-analyses have demonstrated that smoking tobacco is associated with a 2-fold increase in risk of incident schizophrenia (3) or broader psychosis (2). Based on these systematic reviews and our own literature search, we identified eight studies that examined the longitudinal association between tobacco smoking and incident SSD of which six demonstrated a positive association (13–15, 18, 19, 21), one a negative association (16) and the final study showed no association (20). Using the Bradford Hill framework, a causal association between tobacco smoking and onset of SSD is discussed on the basis of strength of association, temporality, dose-response, adjustment for confounding factors, biological plausibility, and consistency of the association.

### Strength

Of the six studies that found a positive association (13–15, 18, 19, 21), five reported moderate to large effect sizes (22) (Tables 2, 3) consistent with a causal relationship (8). Sørensen et al. (15) reported a smaller effect size with a 42% increase in the odds of schizophrenia spectrum disorder in people who smoked cigarettes.

**Table 3:** Assessment of studies against Bradford Hill criteria.

**References**	**Strength of Association**	**Temporality**	**Dose-response**	**Confounding**
Buchy et al. (20)	No Association	Not examined	Not examined	Limited adjustment
Kendler et al. (13)	Smoking cigarettes was associated with a twofold (light smokers) to threefold (heavy smokers) increase in risk of schizophrenia	Yes, longitudinal cohort study.	Yes	Adequately adjusted
McGrath et al. (19)	Smoking cigarettes before age of 15 was associated with a threefold risk of psychosis	Yes, longitudinal cohort study.	Not examined	Adequately adjusted
Mustonen et al. (21)	Smoking ≥10 cigarettes/day was associated with an almost threefold increased risk of psychosis	Yes, longitudinal cohort study.	Yes	Adequately adjusted
Sørensen et al. (15)	Smokers had a 42% increase in risk of incident schizophrenia spectrum disorder	Yes, longitudinal cohort study.	Yes	Adequately adjusted
Weiser et al. (14)	Smoking cigarettes had a twofold risk in incident schizophrenia	Yes, longitudinal cohort study.	Yes	Adequately adjusted
Wium-Andersen et al. (18)	Smoking ≥20 cigarettes/day had a six fold risk of developing schizophrenia	Yes, longitudinal cohort study.	Yes	Adequately adjusted
Zammit et al. (16)	Smoking ≥20 cigarettes/day had a 50% reduction in risk of developing schizophrenia	Yes, longitudinal cohort study.	There was a significant linear trend where smoking decreased the risk of subsequent schizophrenia.	Adequately adjusted

*Green, supportive of causal association; Yellow, not examined or not applicable; Red, no association or negative association*.

### Consistency

Consistency of the association is assessed through multiple studies of independent cohorts confirming the same result. In the eight longitudinal studies, six reported a positive association between tobacco smoking and incident SSD. Of the two which did not report a positive association, one was a case-control study of participants at clinical high risk for psychosis which found that neither tobacco nor cannabis smoking were associated with transition to psychosis. The prevalence of tobacco and cannabis dependence in this cohort was low and the study may have been underpowered to examine the effects of these substances on transition to psychosis. Zammit et al. (16) reported that smoking tobacco was associated with a lower risk of future schizophrenia, and was therefore inconsistent with the main body of research. The overwhelming majority of studies showed a positive relationship fulfilling criteria for consistency.

### Temporality

The six studies that reported a positive association demonstrated a clear temporal relationship with the exposure of tobacco smoking preceding the onset of SSD. Schizophrenia spectrum disorders frequently have an insidious onset with a long prodrome. In order to address this concern, (21), adjusted for prodromal psychotic symptoms at baseline and Kendler et al. (13) accounted for the possible prodrome by conducting a subanalysis restricting the onset of SSD to at least 5 years following initial exposure to tobacco. The relationship between tobacco smoking and onset of schizophrenia was largely attenuated after accounting for the prodrome rendering reverse causality an unlikely explanation for the association between tobacco use and SSD thus suggesting tobacco smoking precedes the illness.

### Dose-response

A dose response between tobacco smoking and incident SSD was reported in five of the six studies reporting a positive association. In three studies (14, 15, 21) a significant linear trend was demonstrated where the risk of SSD increased with the an increase in tobacco smoking. In two studies (13, 18), those who smoked more daily tobacco had an increase in the odds of developing SSD.

### Potential confounders

The relationship between tobacco use and SSD remained significant even after adjusting for factors that might confound the relationship including family socio-economic status, cannabis use (1), parental substance abuse and parental psychosis (23–27). A shared genetic liability was also accounted for in two genetically informed studies (13, 18). Adjustment for confounders attenuated the strength of the association but significance was maintained in all but one study (19), probably due to a lack of power for the analysis. None of the studies adjusted for childhood trauma (28).

### Biological plausibility

Tobacco and tobacco smoke contain almost 5,000 different chemicals. Nicotine is the most important pharmacologically active and psychotogenic compound in tobacco smoke because of its interaction with nicotinic acetylcholine receptors (29). Previous reports on tobacco smoking suggests that nicotine could alter signaling of dopaminergic, cholinergic, and glutamatergic neurotransmitter systems (5, 30) and thus could potentially influence brain development as suggested by studies of adolescent nicotine exposure and neurodevelopmental trajectories (5). Also, excess nicotine intake during early adolescence is associated with abnormal white matter maturation in adults (31), and chronic cigarette smoking has been linked to structural brain changes such as gray matter decreases in the prefrontal cortex, which correspond with areas where functional alterations occur from nicotine exposure (32).

Furthermore, recent evidence suggest that adolescent nicotine use could have persistent effects on nicotine receptor responsiveness, which results in the strengthening of negative emotional changes and alterations in cognitive functioning (5).

### Alternative explanations

There are other explanations for the positive association between tobacco smoking and SSD. Individuals who develop schizophrenia are more likely to have externalizing symptoms in childhood and adolescence (33, 34) and children with externalizing symptoms are more likely to smoke tobacco during adolescence (35). There may be unmeasured confounding. None of the studies adjusted for childhood trauma, a well-established risk factor for SSD (1, 28) and for tobacco use (36). Similarly there was no adjustment for prenatal tobacco smoking exposure which is associated with both an increased risk of smoking in adolescence (37) and an increased risk of schizophrenia even after adjusting for life time smoking (3, 38). Furthermore, recent studies have suggested bidirectional associations by revealing single nucleotide polymorphisms associated with nicotine dependence (CHRNA5) that are also associated with schizophrenia (39, 40).

## Limitations

Each study included in this review is observational in methodology, and the majority of cohort studies included had significant attrition. Participants who are most likely to be lost to follow up are more likely to be socioeconomically disadvantaged and be at increased risk of both tobacco smoking and mental illness. Therefore, it is unlikely that attrition would significantly affect reported associations. Measurement of tobacco smoking has been measured via self-report or by interview, generally at one point in time and often retrospectively recalled. Only one study measured the long-term smoking exposure prior the psychotic illness using pack-years (18) which provides a more precise measurement of tobacco smoke exposure. Further, no studies have used biological markers for tobacco smoking such as expired air carbon monoxide (41) or serum cotinine measurement (42). These limitations are inherent to large cohort and registry studies and are difficult to overcome. Finally, as two recent systematic reviews had been published on this topic, we relied on these to identify the studies included in this review rather than replicating the searches in these studies.

### Implications

Given tobacco is known to have widespread adverse health outcomes and governments around the world are adopting policies to reduce tobacco smoking, why is it important to clarify if smoking tobacco has a causal role in the onset of SSD? The first reason is that better understanding the aetiopathogenesis of SSD will inform our knowledge of this syndrome which may lead to better treatments. The second, a much more urgent consideration is the growing availability of electronic (e) cigarettes. These have been developed as a safer alternative to cigarettes by enabling nicotine use without the exposure to carcinogenic chemicals associated with smoking tobacco.

However, there is growing use of e-cigarettes and other nicotine products by adolescents (6) and it is acknowledged that the health effects of e-cigarettes on youth are not fully understood (43). In addition to tobacco and cannabis, there is now evidence that adolescents who use inhalants are at increased risk of psychotic disorders (44) suggesting that adolescence is the developmental period where adverse neuropsychiatric outcomes from psychoactive substances are most likely to occur. There is substantial though not conclusive evidence that the association between tobacco smoking and SSD is causal and may well be a result of the effects of nicotine on multiple neurotransmitter systems. Therefore, policy makers must be cautious when developing regulations for the availability of e-cigarettes, nicotine replacement therapy products and smokeless tobacco. Similarly, health practitioners who recommend e-cigarettes or smokeless tobacco products as a safe alternative to smoking need to consider the findings of the studies identified in this review, especially when providing advice to adolescents.

It is essential that future well designed observational studies are undertaken examining the risk of SSDs in those who use e-cigarettes, particularly in adolescence. A major challenge is the low prevalence of SSD. Recruiting samples large enough to examine the association between e-cigarettes and SSD will take many years. Previous longitudinal research has shown positive associations between cannabis, tobacco and alcohol use and psychotic experiences (PE) which are proxy markers for psychosis risk. PE have the advantage of being higher in prevalence compared to SSD thereby reducing the required sample size to identify associations. Schizophrenia endophenotypes may also have a role to inform the association between nicotine exposure through e-cigarettes and risk of SSD. Previous research has shown that smoking tobacco modulates the association between polymorphisms of transcription factor 4 and reduced sensory gating, an endophenotype of schizophrenia suggesting that the smoking of tobacco might play a role in early information processing deficits in schizophrenia (45). Use of research paradigms such as PE and endophenotypes PE would expedite research into the association between e-cigarette use and SSD risk. Further research is urgently needed to determine if nicotine is causally associated with incident SSD. In the interim, it is important that policy makers consider the available evidence between tobacco smoking and risk of schizophrenia when evaluating the potential health consequences that might arise from community access to e-cigarettes.

## Author contributions

JS and AM planned the review. LM conducted the initial literature search and JS and LM assessed papers for suitability for inclusion. JS and LM reviewed all the papers and assessed them for quality. JS, LM, and AM wrote the first draft of the manuscript and all authors contributed to further drafts. All authors reviewed and approved the final draft.

### Conflict of interest statement

The authors declare that the research was conducted in the absence of any commercial or financial relationships that could be construed as a potential conflict of interest.

## References

[1] Belbasis L Köhler CA Stefanis N Stubbs B Os Jv Vieta E. Risk factors and peripheral biomarkers for schizophrenia spectrum disorders: an umbrella review of meta-analyses. Acta Psychiatr Scand. (2018) 137:88–97. 10.1111/acps.12847 29288491

[2] Gurillo P Jauhar S Murray RM MacCabe JH. Does tobacco use cause psychosis? Systematic review and meta-analysis. Lancet Psychiatry (2015) 2:718–25. 10.1016/S2215-0366(15)00152-2 26249303 PMC4698800

[3] Hunter A Murray R Asher L Leonardi-Bee J. The effects of tobacco smoking, and prenatal tobacco smoke exposure, on risk of schizophrenia: a systematic review and meta-analysis. Nicotine Tob Res. (2018). 10.1093/ntr/nty160. [Epub ahead of print]. 30102383

[4] Collaborators GT Smoking prevalence and attributable disease burden in 195 countries and territories, 1990-2015: a systematic analysis from the Global Burden of Disease Study 2015. Lancet (2017) 389:1885–906. 10.1016/s0140-6736(17)30819-x 28390697 PMC5439023

[5] Smith RF McDonald CG Bergstrom HC Ehlinger DG Brielmaier JM. Adolescent nicotine induces persisting changes in development of neural connectivity. Neurosci Biobehav Rev (2015) 55:432–43. 10.1016/j.neubiorev.2015.05.019 26048001

[6] Perikleous EP Steiropoulos P Paraskakis E Constantinidis TC Nena E. E-cigarette use among adolescents: an overview of the literature and future perspectives. Front Public Health (2018) 6:86. 10.3389/fpubh.2018.00086 29632856 PMC5879739

[7] Fedak KM Bernal A Capshaw ZA Gross S. Applying the bradford hill criteria in the 21st century: how data integration has changed causal inference in molecular epidemiology. Emerg Themes Epidemiol. (2015) 12:14. 10.1186/s12982-015-0037-4 26425136 PMC4589117

[8] Hill AB The environment and disease: association or causation? Proc R Soc Med. (1965) 58:295–300. 14283879 10.1177/003591576505800503 PMC1898525

[9] Wells G Shea B O'Connell D Peterson J Welch V Losos M The Newcastle-Ottawa Scale (NOS) for Assessing the Quality of Nonrandomised Studies in Meta-Analyses. Ottawa: The Ottawa Hospital (2018).

[10] Hindocha C Shaban NDC Freeman TP Das RK Gale G Schafer G. Associations between cigarette smoking and cannabis dependence: a longitudinal study of young cannabis users in the United Kingdom. Drug Alcohol Depend. (2015) 148:165–71. 10.1016/j.drugalcdep.2015.01.004 25622777 PMC4337852

[11] Badiani A Boden JM De Pirro S Fergusson DM Horwood LJ Harold GT. Tobacco smoking and cannabis use in a longitudinal birth cohort: evidence of reciprocal causal relationships. Drug Alcohol Depend. (2015) 150:69–76. 10.1016/j.drugalcdep.2015.02.015 25759089

[12] Weinberger AH Platt J Copeland J Goodwin RD. Is cannabis use associated with increased risk of cigarette smoking initiation, persistence, and relapse? longitudinal data from a representative sample of US adults. J Clin Psychiatry (2018) 79:17m11522. 10.4088/JCP.17m11522 29570966 PMC6355334

[13] Kendler KS Lönn SL Sundquist J Sundquist K. Smoking and schizophrenia in population cohorts of swedish women and men: a prospective co-relative control study. Am J Psychiatry (2015) 172:1092–100. 10.1176/appi.ajp.2015.15010126 26046339 PMC4651774

[14] Weiser M Reichenberg A Grotto I Yasvitzky R Rabinowitz J Lubin G. Higher rates of cigarette smoking in male adolescents before the onset of schizophrenia: a historical-prospective cohort study. Am J Psychiatry (2004) 161:1219–23. 10.1176/appi.ajp.161.7.1219 15229054

[15] Sørensen HJ Mortensen EL Reinisch JM Mednick SA. A prospective study of smoking in young women and risk of later psychiatric hospitalization. Nordic J Psychiatry (2011) 65:3–8. 10.3109/08039481003786386 20429749

[16] Zammit S Allebeck P Dalman C Lundberg I Hemmingsson T Lewis G. Investigating the association between cigarette smoking and schizophrenia in a cohort study. Am J Psychiatry (2003) 160:2216–21. 10.1176/appi.ajp.160.12.2216 14638593

[17] Riala K Hakko H Isohanni M Pouta A Räsänen P. Is initiation of smoking associated with the prodromal phase of schizophrenia? J Psychiatry Neurosci. (2005) 30:26–32. 15644994 PMC543837

[18] Wium-Andersen MK Orsted DD Nordestgaard BG Tobacco smoking is causally associated with antipsychotic medication use and schizophrenia, but not with antidepressant medication use or depression. Int J Epidemiol. (2015) 44:566–77. 10.1093/ije/dyv090 26054357

[19] McGrath JJ Alati R Clavarino A Williams GM Bor W Najman JM. Age at first tobacco use and risk of subsequent psychosis-related outcomes: a birth cohort study. Aust N Z J Psychiatry (2016) 50:577–83. 10.1177/0004867415587341 25991762

[20] Buchy L Cadenhead KS Cannon TD Cornblatt BA McGlashan TH Perkins DO. Substance use in individuals at clinical high risk of psychosis. Psychol Med. (2015) 45:2275–84. 10.1017/S0033291715000227 25727300 PMC8182984

[21] Mustonen A Ahokas T Nordström T Murray GK Mäki P Jääskeläinen E. Smokin‘ hot: adolescent smoking and the risk of psychosis. Acta Psychiatr Scand. (2018) 138:5–14. 10.1111/acps.12863 29457219

[22] Rosenthal JA Qualitative descriptors of strength of association and effect size. J Soc Serv Res. (1996) 21:37–59. 10.1300/J079v21n04_02

[23] McAdams TA Neiderhiser JM Rijsdijk FV Narusyte J Lichtenstein P Eley TC. Accounting for genetic and environmental confounds in associations between parent and child characteristics: a systematic review of children-of-twins studies. Psychol Bull. (2014) 140:1138–73. 10.1037/a0036416 24749497

[24] Niemi-Pynttäri JA Sund R Putkonen H Vorma H Wahlbeck K Pirkola SP. Substance-induced psychoses converting into schizophrenia: a register-based study of 18,478 Finnish inpatient cases. J Clin Psychiatry (2013) 74:e94–9. 10.4088/JCP.12m07822 23419236

[25] Patrick ME Wightman P Schoeni RF Schulenberg JE. Socioeconomic status and substance use among young adults: a comparison across constructs and drugs. J Stud Alcohol Drugs (2012) 73:772–82. 10.15288/jsad.2012.73.772 22846241 PMC3410945

[26] Radhakrishnan R Wilkinson ST D'Souza DC. Gone to pot - a review of the association between cannabis and psychosis. Front Psychiatry (2014) 5:54. 10.3389/fpsyt.2014.00054 24904437 PMC4033190

[27] Starzer MSK Nordentoft M Hjorthøj C. Rates and predictors of conversion to schizophrenia or bipolar disorder following substance-induced psychosis. Am J Psychiatry (2018) 175:343–50. 10.1176/appi.ajp.2017.17020223 29179576

[28] Varese F Smeets F Drukker M Lieverse R Lataster T Viechtbauer W. Childhood adversities increase the risk of psychosis: a meta-analysis of patient-control, prospective- and cross-sectional cohort studies. Schizophr Bull. (2012) 38:661–71. 10.1093/schbul/sbs050 22461484 PMC3406538

[29] Thielen A Klus H Müller L. Tobacco smoke: unraveling a controversial subject. Exp Toxicol Pathol. (2008) 60:141–56. 10.1016/j.etp.2008.01.014 18485684

[30] Davis J Eyre H Jacka FN Dodd S Dean O McEwen S. A review of vulnerability and risks for schizophrenia: Beyond the two hit hypothesis. Neurosci Biobehav Rev. (2016) 65:185–94. 10.1016/j.neubiorev.2016.03.017 27073049 PMC4876729

[31] Gogliettino AR Potenza MN Yip SW. White matter development and tobacco smoking in young adults: a systematic review with recommendations for future research. Drug Alcohol Depend. (2016) 162:26–33. 10.1016/j.drugalcdep.2016.02.015 26948756 PMC4833590

[32] Sutherland MT Riedel MC Flannery JS Yanes JA Fox PT Stein EA. Chronic cigarette smoking is linked with structural alterations in brain regions showing acute nicotinic drug-induced functional modulations. Behav Brain Funct. (2016) 12:16. 10.1186/s12993-016-0100-5 27251183 PMC4890474

[33] Scott J Martin G Welham J Bor W Najman J O'Callaghan M. Psychopathology during childhood and adolescence predicts delusional-like experiences in adults: a 21-year birth cohort study. Am J Psychiatry (2009) 166:567–74. 10.1176/appi.ajp.2008.08081182 19339357

[34] Matheson SL Shepherd AM Laurens KR Carr VJ. A systematic meta-review grading the evidence for non-genetic risk factors and putative antecedents of schizophrenia. Schizophr Rese. (2011) 133:133–42. 10.1016/j.schres.2011.09.020 21999904

[35] Miettunen J Murray GK Jones PB Maki P Ebeling H Taanila A. Longitudinal associations between childhood and adulthood externalizing and internalizing psychopathology and adolescent substance use. Psychol Med. (2014) 44:1727–38. 10.1017/S0033291713002328 24028974

[36] Norman RE Byambaa M De R Butchart A Scott J Vos T. The long-term health consequences of child physical abuse, emotional abuse, and neglect: a systematic review and meta-analysis. PLoS Med. (2012) 9:e1001349. 10.1371/journal.pmed.1001349 23209385 PMC3507962

[37] Niemela S Raisanen A Koskela J Taanila A Miettunen J Ramsay H. The effect of prenatal smoking exposure on daily smoking among teenage offspring. Addiction (2017) 112:134–43. 10.1111/add.13533 27444807

[38] Niemela S Sourander A Surcel HM Hinkka-Yli-Salomaki S McKeague IW Cheslack-Postava K. Prenatal nicotine exposure and risk of schizophrenia among offspring in a national birth cohort. Am J Psychiatry (2016) 173:799–806. 10.1176/appi.ajp.2016.15060800 27216261

[39] Chen J Bacanu SA Yu H Zhao Z Jia P Kendler KS. Genetic relationship between schizophrenia and nicotine dependence. Sci Rep. (2016). 6:25671. 10.1038/srep25671. 27164557 PMC4862382

[40] Hartz SM Horton AC Hancock DB Baker TB Caporaso NE Chen LS. Genetic correlation between smoking behaviors and schizophrenia. Schizophr Res. (2018) 194:86–90. 10.1016/j.schres.2017.02.022 28285025 PMC5811408

[41] Jarvis MJ Russell MA Saloojee Y. Expired air carbon monoxide: a simple breath test of tobacco smoke intake. Br Med J. (1980) 281:484–5. 7427332 10.1136/bmj.281.6238.484 PMC1713376

[42] Vartiainen E Seppala T Lillsunde P Puska P. Validation of self reported smoking by serum cotinine measurement in a community-based study. J Epidemiol Commun Health (2002) 56:167–70. 10.1136/jech.56.3.167 11854334 PMC1732104

[43] National Academies of Sciences Engineering and Medicine; Health and Medicine Division; Board on Population Health and Public Health Practice; Committee on the Review of the Health Effects of Electronic Nicotine Delivery Systems Public Health Consequences of E-Cigarettes. In: Eaton DL Kwan LY Stratton K, editors. Washington, DC: National Academies Press (US) (2018). 29894118

[44] Mustonen A Niemela S McGrath JJ Murray GK Nordstrom T Maki P. Adolescent inhalant use and psychosis risk-a prospective longitudinal study. Schizophr Res. (2018). 10.1016/j.schres.2018.05.013. [Epub ahead of print]. 29958751

[45] Quednow BB Brinkmeyer J Mobascher A Nothnagel M Musso F Grunder G. Schizophrenia risk polymorphisms in the TCF4 gene interact with smoking in the modulation of auditory sensory gating. Proc Natl Acad Sci USA. (2012) 109:6271–6. 10.1073/pnas.1118051109 22451930 PMC3341057

