# Supplementary material for: Evidence of a Causal Relationship Between Smoking Tobacco and Schizophrenia Spectrum Disorders
Source: Front Psychiatry. 2018 Nov 20;9:607. doi: 10.3389/fpsyt.2018.00607 (PMC6255982; doi:10.3389/fpsyt.2018.00607)
Supplement: Supplementary file 1 [file Table_1.DOCX]

Supplementary Table 1 Newcastle-Ottawa Quality Assessment Scale used for **cohort** studies for this review

Note: A study can be awarded a maximum of one star (*) for each numbered item within the Selection and Outcome categories. A maximum of two stars can be given for Comparability.

| **Selection** |
| --- |
| **1. Representativeness of the exposed cohort**  a) truly representative of tobacco smokers in the community *  b) somewhat representative of the average tobacco smokers in the community (i.e. not entire tobacco smoking population, but large sample size) *  c) selected group of users (e.g help seekers, university students, online recruitment)  d) no description of the derivation of the cohort |
| **2. Selection of the non-exposed (non-tobacco smoking) cohort**  a) drawn from the same community as the tobacco smoking cohort *  b) drawn from a different source  c) no description of the derivation of the non-tobacco smoking cohort |
| **3. Ascertainment of exposure**  a) Biological marker of tobacco use (e.g. Expired Air Carbon Monoxide) *  b) Structured interview where interviewer is blind to smoking (exposure) status *  c) written self-report  d) no description |
| **4. Demonstration that schizophrenia or psychosis was not present at start of study**  a) yes, smoking status was ascertained prior to schizophrenia or psychosis diagnosis *  b) no |
| **Comparability** |
| **5. Comparability of cohorts on the basis of the design or analysis**  a) study controls for cannabis use *  b) study controls for additional factors including sex, age and socioeconomic status * |
| **Outcome** |
| **6. Assessment of outcome**  a) Structured interview where interviewer is blind to schizophrenia or psychosis (outcome) status *  b) Clinical diagnosis of schizophrenia or psychosis (attained through medical records) *  c) self-report  d) no description |
| **7. Was follow-up long enough for outcomes to occur**  a) yes (>12 months) *  b) no (<12 months) |
| **8. Adequacy of follow-up of cohorts**  a) complete follow up - all subjects accounted for *  b) subjects lost to follow up unlikely to introduce bias - small number lost (<20%) or description provided of those lost *  c) follow up rate <80% (i.e. >20% lost) and no description of those lost  d) no statement |
