# Supplementary material for: Evidence of a Causal Relationship Between Smoking Tobacco and Schizophrenia Spectrum Disorders
Source: Front Psychiatry. 2018 Nov 20;9:607. doi: 10.3389/fpsyt.2018.00607 (PMC6255982; doi:10.3389/fpsyt.2018.00607)
Supplement: Supplementary file 2 [file Table_2.DOCX]

**Table 2. Explanation of Bradford Hill Criteria**

| Criteria | Explanation of how it relates to this study | Use in the current study |
| --- | --- | --- |
| 1. Strength of Association | Statistically significant and practically significant results. | Included |
| 2. Consistency of Association | Results supported by different studies, using different techniques, in different study populations/ecological areas. | Included |
| 3. Specificity of Association | Specificity of the exposure to lead to the outcome of interest, and not a number of exposures leading to the outcome of interest. | Not included |
| 4. Temporality of Association | Evidence that the exposure precedes the outcome. | Included |
| 5. Dose-Response Relationship | Evidence that there is a gradient of risk associated with the degree of exposure, or a threshold effect. | Included |
| 6. Biological Plausibility | Plausibility of the relationship based on review of biological evidence. | Included |
| 7. Coherence | Coherence of the association with current research. | Not included |
| 8. Experiment | A counter-factual approach, whereby removal of the exposure prevents the outcome from occurring. | Not included |
| 9. Analogy | Evidence of similar exposures causing similar outcomes, or knowledge of established risk factors with similar causal routes. | Not included |
|  |  |  |
| *Additional Criteria:* Confounding | Inclusion of known risk factors of the outcome in the analysis, adjustment for confounding and evidence that the association between exposure and outcome is not due to confounding | Included |
